# Further Elucidation of the Argonaute and Dicer Protein Families in the Model Grass Species *Brachypodium distachyon*

**DOI:** 10.3389/fpls.2019.01332

**Published:** 2019-10-22

**Authors:** Ena Šečić, Silvia Zanini, Karl-Heinz Kogel

**Affiliations:** Institute of Phytopathology, Centre for BioSystems, Land Use and Nutrition, Justus Liebig University, Giessen, Germany

**Keywords:** protein, structure, prediction, RNAi, Argonaute, Dicer, *Brachypodium*

## Abstract

RNA interference (RNAi) is a biological process in which small RNAs regulate gene silencing at the transcriptional or posttranscriptional level. The trigger for gene silencing is double-stranded RNA generated from an endogenous genomic locus or a foreign source, such as a transgene or virus. In addition to regulating endogenous gene expression, RNAi provides the mechanistic basis for small RNA-mediated communication between plant hosts and interacting pathogenic microbes, known as cross-kingdom RNAi. Two core protein components, Argonaute (AGO) and Dicer (DCL), are central to the RNAi machinery of eukaryotes. Plants encode for several copies of *AGO* and *DCL* genes; in *Arabidopsis thaliana*, the AGO protein family contains 10 members, and the DCL family contains four. Little is known about the conservation and specific roles of these proteins in monocotyledonous plants, which account for the most important food staples. Here, we utilized *in silico* tools to investigate the structure and related functions of AGO and DCL proteins from the model grass *Brachypodium distachyon*. Based on the presence of characteristic domains, 16 BdAGO- and 6 BdDCL-predicted proteins were identified. Phylogenetic analysis showed that both protein families were expanded in *Brachypodium* as compared with *Arabidopsis*. For BdDCL proteins, both plant species contain a single copy of DCL1 and DCL4; however, *Brachypodium* contains two copies each of DCL2 and DCL3. Members of the BdAGO family were placed in all three functional clades of AGO proteins previously described in *Arabidopsis*. The greatest expansion occurred in the AtAGO1/5/10 clade, which contains nine BdAGOs (BdAGO5/6/7/9/10/11/12/15/16). The catalytic tetrad of the AGO P-element-induced wimpy testis domain (PIWI), which is required for endonuclease activity, is conserved in most BdAGOs, with the exception of BdAGO1, which lacks the last D/H residue. Three-dimensional modeling of BdAGO proteins using tertiary structure prediction software supported the phylogenetic classification. We also predicted a provisional interactome network for BdAGOs, their localization within the cell, and organ/tissue-specific expression. Exploring the specifics of RNAi machinery proteins in a model grass species can serve as a proxy for agronomically important cereals such as barley and wheat, where the development of RNAi-based plant protection strategies is of great interest.

## Introduction

RNA interference (RNAi) is a regulatory mechanism utilized by most eukaryotes for endogenous gene silencing and protection against mobile repetitive sequences, transposons, and viruses (Fire et al., 1998; Wilson and Doudna, 2013). In contrast to transcriptional gene silencing (TGS), which results in the methylation of DNA and/or histones, posttranscriptional gene silencing (PTGS) operates by transcript degradation or translation inhibition. Selection of the target for silencing is governed by sequence complementarity between a single-stranded small RNA (sRNA) and the target RNA. Beyond its native role, the RNAi machinery has been exploited for developing novel plant protection strategies based on double-stranded (ds)RNA applications. Delivery of artificial dsRNA through transgene expression [host-induced gene silencing (HIGS)] or exogenous application [spray-induced gene silencing (SIGS)] was proven effective against fungal pathogens (Nowara et al., 2010; Koch et al., 2013; Koch et al., 2016; Wang et al., 2016), nematodes (Dutta et al., 2015), insects (Coleman et al., 2014; Abdellatef et al., 2015; Head et al., 2017), and parasitic plants (Tomilov et al., 2008; for review, see Andrade and Hunter, 2016; Cai et al., 2018). A recent discovery revealed that RNAi also is involved in natural cross-kingdom RNA communication (ckRNAi), where sRNA molecules function as mediators that are exchanged bidirectionally between a host plant and a microbial pathogen to silence their target transcripts and impact the outcome of the plant–pathogen interaction (Weiberg et al., 2013; Zhang et al., 2016; Wang et al., 2017a; Wang et al., 2017b).

Regardless of which RNAi-based process or application is involved, evolutionarily conserved protein components, including Dicer [termed Dicer-like (DCL) in plants] and Argonaute (AGO), play key roles in dsRNA processing. Dicers and DCLs are RNase III endonucleases that process exogenously supplied or endogenously generated ds- or hairpin (hp)-containing RNA precursors into various species of dsRNAs, commonly 21–24 nucleotides (nt) in length. These sRNAs are then loaded onto specific AGO proteins, which are components of the RNA-induced silencing complex (RISC). The loaded sRNA is processed into a single-stranded sRNA molecule, which then guides the RISC to complementary targets in the cytoplasm or nucleus. Depending on the biological context, target recognition leads to PTGS *via* RNA degradation, which may be mediated by the AGO protein’s slicer activity, or inhibition of translation, or to TGS *via* genomic DNA and/or histone methylation (Carthew and Sontheimer, 2009; Poulsen et al., 2013; Borges and Martienssen, 2015; Fang and Qi, 2016).

Phylogenetic analysis of genes belonging to the Dicer family suggests that they arose early in the evolution of eukaryotes and that their duplication and diversification correlated with the development of multicellularity and the need for complex gene regulation (Mukherjee et al., 2013). In plants, the structure and function of DCL proteins have been investigated most intensively in *Arabidopsis*, which expresses four DCLs (Schauer et al., 2002). The domain architecture of these proteins, like that of other eukaryotic Dicers, generally consists of an amino-terminal DEXDc and helicase-C (HELICc) domain, which are thought to mediate processive movement along a dsRNA, a dicer-dimer (heterodimerization) domain that facilitates binding with protein partners (Qin et al., 2010), a P-element-induced wimpy testis (PIWI)–Argonaute–Zwille (PAZ) domain, which binds the 3’ end of the dsRNA, two RIBOc (ribonuclease III family) domains and at least one dsRNA-binding motif (DSRM) domain at the C terminus (Schauer et al., 2002; Mukherjee et al., 2013; Bologna and Voinnet, 2014; Song and Rossi, 2017).

Analyses of *Arabidopsis* mutants revealed that the four DCLs generate different types of sRNAs, although some functional redundancy was observed (Gasciolli et al., 2005; Bologna and Voinnet, 2014; Borges and Martienssen, 2015). AtDCL1 produces microRNAs (miRNAs), a class of sRNAs that regulates endogenous gene expression via PTGS (Bartel, 2004). The remaining AtDCLs generate various subclasses of small interfering RNAs (siRNAs), including i) natural-antisense-transcript (nat)-siRNAs generated by AtDCL2 (Borsani et al., 2005), ii) trans-activating (ta)-siRNAs produced by AtDCL4 (Dunoyer et al., 2005), and iii) TGS-related 24-nt siRNAs generated by AtDCL3, which are responsible for silencing transposons and other repeated DNA sequences (Xie et al., 2004).

Despite the diversity of sRNAs, their association with AGO proteins and the RISC complex is a common feature. AGO proteins were named after the tube-shaped leaves of *Arabidopsis ago1* mutants, which resemble the tentacles of the pelagic octopus, *Argonauta argo* (Bohmert et al., 1998). AGO proteins are highly conserved in nature, although the size of this family varies substantially between species (Höck and Meister, 2008; Zhang et al., 2015; Fang and Qi, 2016; You et al., 2017).

AGOs have a high level of structure and domain conservation between the prokaryotic and eukaryotic variants, even when the biological function clearly differs (Willkomm et al., 2015). Several prokaryotic (Wang et al., 2009; Liu et al., 2018) and eukaryotic (Lingel et al., 2003; Lingel et al., 2004; Boland et al., 2011) complete AGO structures or individual domains have been crystallographically resolved. Several human AGO proteins have been crystallized, namely, AGO2 in complex with a miRNA (Elkayam et al., 2012), AGO1 (Faehnle et al., 2013), and AGO3 (Park et al., 2017), showing that the AGO activity is dependent on conservation of active site residues and their interaction with other protein regions. The structures of AtAGO proteins have been partly resolved, especially the middle (MID) domain of AtAGO1, AtAGO2, and AtAGO5 (Frank et al., 2012; Zha et al., 2012). Functional domains characteristic of all AGO proteins, including the *Arabidopsis* AGOs, are the PAZ, MID, and PIWI domains governing the binding of sRNA ends and the slicer activity (Höck and Meister, 2008; Frank et al., 2012).

In *Arabidopsis*, 10 different AGOs have been identified. Phylogenetic analyses have divided them into three clades, comprising AGO1/5/10, AGO2/3/7, and AGO4/6/8/9 (Vaucheret, 2008). The different clades contain AGOs that mediate PTGS or TGS after they load specific types of sRNAs, which are selected based on length and identity of the 5’ nt (Bologna and Voinnet, 2014; Zhang et al., 2015; Fang and Qi, 2016). For example, AtAGO1 is involved in endogenous developmental regulation by miRNAs (Vaucheret et al., 2004), antiviral defense (alongside AtAGO2, Harvey et al., 2011), as well as bidirectional ckRNAi (Weiberg et al., 2013). AtAGO4 and AtAGO6 are involved in DNA and histone methylation (Zilberman et al., 2003; Zheng et al., 2007). AtAGO9 is known to be involved in female gametogenesis (Olmedo-Monfil et al., 2010), while AtAGO10 competes with AtAGO1 for sRNA loading in regulation of shoot apical meristem development (Zhu et al., 2011). AtAGO7 plays a role in defense against viruses (Qu et al., 2008).

In comparison to *Arabidopsis*, little is known about the RNAi machinery components in monocots. The number of AGO proteins is expanded in cereals, as there are 17 AGOs in maize (Zea mays) and 19 in rice (Oryza sativa; Fang and Qi, 2016; Mirzaei et al., 2014; Patel et al., 2018). The copy number of DCL2 and/or DCL3 genes also differs between monocot species and *Arabidopsis*. Six predicted DCLs were identified in rice, while five were identified in maize, wheat, and barley (Margis et al., 2006). Furthermore, the DCL3b gene has diverged significantly from its DCL3a paralog (Margis et al., 2006) and, thus, is considered a distinct, monocot-specific class of Dicer, termed DCL5 (Fukudome and Fukuhara, 2017; Borges and Martienssen, 2015).

Cereals are major staple crops worldwide; however, a plethora of pathogens and pests threaten their production (Savary et al., 2012). Recent efforts to develop environmentally friendly plant protection strategies have demonstrated that HIGS and SIGS can be used in major cereal crops, such as barley and wheat, to control necrotrophic fungi (Koch et al., 2013; Koch et al., 2016; Koch et al., 2018) and aphid pests (Abdellatef et al., 2015). Developing a better understanding of the cereal AGO and DCL protein family members and their specific functions is a prerequisite for clarifying the mechanisms undergirding RNAi-mediated plant protection. Here, we use the model species for temperate grass plants, *Brachypodium distachyon* (*Brachypodium*), to investigate cereal AGO and DCL proteins. *Brachypodium* is self-fertile, has a small genome (∼272 Mb), a short life cycle, and established transformation protocols (Vogel et al., 2006). The commonly used diploid inbred line Bd21 is fully sequenced (The International Brachypodium Initiative, 2010). In addition, literature data reveal strong responsiveness of *Brachypodium* sRNA pools to abiotic stress, suggesting that the RNAi machinery is sensitive to environmental changes (Wang et al., 2015).

Based on genomic database searches and *in silico* analysis, we identified six BdDCL proteins, as well as 16 previously reported AGO protein sequences in *Brachypodium* (Mirzaei et al., 2014). Since the structure of proteins closely relates to function and thus can serve as an indication of interaction patterns and redundancy in large protein families, we especially looked into domain structure conservation in *Brachypodium* relative to *Arabidopsis*. Similar to the protein structure and interactome analysis applied in Secic et al. (2015), we subjected Bd AGO and DCL proteins to a series of *in silico* analysis steps. The focus of this study is the structures and related functions of the AGO-like and DCL proteins of *B. distachyon*, with special regard to analysis of the phylogeny and three-dimensional (3D) structure modeling of the AGO family, as compared with the more familiar *Arabidopsis thaliana* AGO protein family. Given that the At AGO1/5/10 clade contains proteins involved in ckRNAi, we were especially interested to define the BdAGO proteins that are structurally most related to this clade and thus potentially have a key function in plant immunity and RNAi-based plant protection.

## Materials and Methods

### Acquisition of Sequences and Database Search

AGO and DCL protein sequences corresponding to the primary transcripts of specific genes were acquired by searching the Plant Comparative Genomics portal Phytozome 12 (Goodstein et al., 2012) *B. distachyon* v3.1 database (The International Brachypodium Initiative, 2010). Proteins whose domain architecture resembled those of *Arabidopsis* AGO and DCL proteins were considered. The *Arabidopsis* AGO and DCL protein sequences were taken from The *Arabidopsis* Information Resource database (Rhee et al., 2003; Berardini et al., 2015). Information on resolved protein structures was acquired from the Research Collaboratory for Structural Bioinformatics Protein Data Bank (Berman et al., 2000; Burley et al., 2018). The Brachypodium eFP Browser (Sibout et al., 2017; Winter et al., 2007) was used to assess the expression of transcripts corresponding to proteins involved in this study, based on the expression atlas detailing different organs and developmental stages.

### Phylogenetic Analysis, Interactome Analysis, and Localization

The phylogenetic analysis and tree rendering were done by the Phylogeny.fr web server (Dereeper et al., 2008; Dereeper et al., 2010). The operational sequence is composed of MUSCLE 3.8.31 (Edgar, 2004) for alignment with default settings, Gblocks 0.91b for removal of ambiguous regions (Castresana, 2000), PhyML 3.1/3.0 aLRT for phylogeny (Guindon and Gascuel, 2003; Anisimova and Gascuel, 2006), based on maximum likelihood, and TreeDyn 198.3 for graphical representation (Chevenet et al., 2006). Multiple sequence alignment (MSA) was done using Clustal Omega at European Molecular Biology Laboratory—European Bioinformatics Institute (Sievers et al., 2011; Goujon et al., 2010) and the conserved residues and domains visualized by the Mview multiple alignment viewer (Brown et al., 1998). Pairwise sequence alignments were done using EMBOSS Needle (Rice et al., 2000), utilizing the Needleman–Wunsch algorithm for global alignment. Domain search was conducted using Simple Modular Architecture Research Tool (SMART) in normal SMART mode (Schultz et al., 1998; Letunic and Bork, 2017) and visualized with the Illustrator for Biological Sequences (IBS) online illustrator (Liu et al., 2015). Prediction of protein location was done using the plant subcellular localization integrative predictor (PSI), which shows an integrative result based on the output of an 11-member predictor community (Liu et al., 2013). Prediction of the interactome was done using the STRING database of protein–protein associations, while searching by protein sequence (Szklarczyk et al., 2019). Resulting associations/possible interactions that originate from text mining have been excluded, and the results show only associations supported by co-expression and/or experimental data.

### Three-Dimensional Structure Modeling and Validation

SWISS-MODEL, a homology-based modeling software available at the ExPASy web server (Waterhouse et al., 2018), and CPHmodels 3.2 protein homology modeling server (Nielsen et al., 2010) were both used for 3D structure prediction from the sequence data. BLAST (Camacho et al., 2009) and HHBlits (Remmert et al., 2011) template search through the SWISS-MODEL template library was done and the models built using ProMod3 (Waterhouse et al., 2018) and the target-template alignment.

QMEAN, used for validation of the predicted 3D structures, is a scoring function that considers single residues and the global model, delivering an estimation of absolute quality of the prediction (Benkert et al., 2011). In order to check the stereochemical quality of predicted structures, we used the PROCHECK program (Morris et al., 1992; Laskowski et al., 1993). One of the stereochemical parameters considered is the fitness of the model in a Ramachandran plot, which maps the allowed backbone dihedral angles of amino acids (aa) in a protein structure (Ramachandran et al., 1963). Further on, we used the WHATCHECK software (Hooft et al., 1996) to calculate the Ramachandran Z-score, which compares the quality of the query structure to structures with high confidence (Hooft et al., 1997). Lastly, we used the dDFIRE/DFIRE2 energy calculation (Yang and Zhou, 2008) to calculate free energy scores for our structure predictions.

PyMOL (The Py-MOL Molecular Graphics System) was used for visualization of the predicted structures (Schrödinger, 2010, Open-Source PyMOL 1.3).

## Results

### Argonaute and Dicer Protein Families Are Expanded in *Brachypodium* Relative to *Arabidopsis*

To identify AGO and DCL proteins, the *B. distachyon* v3.1 database (The International Brachypodium Initiative, 2010) was searched for transcripts whose encoded proteins contain the characteristic domain architecture of each protein family. The accession numbers of the acquired sequences, the names assigned to the corresponding BdAGO proteins, the location of the encoding genes, and a description of the primary transcripts are shown in **Table 1**. The naming convention is similar to that used by Mirzaei et al. (2014) for 16 AGO proteins identified by primary transcripts in the *B. distachyon* Bd21 v3.1 annotation (The International Brachypodium Initiative, 2010). Our search for BdDCL candidates within the Bd21 v3.1 database revealed nine sequences. Clear lack of functional domains or insufficient length of the deduced aa sequence reduced the number of putative *DCL* genes to six (Bradi1g15440, Bradi1g77087, Bradi2g23187, Bradi5g15337, Bradi1g21030, and Bradi3g29287). Accession numbers and assigned names for the encoded BdDCLs are shown in **Table 2**. The putative AtAGO and AtDCL protein sequences were downloaded from The *Arabidopsis* Information Resource database (**Table S1**) and included in the MSA and phylogenetic analysis.

**Table 1:** Assigned names and accession numbers of BdAGO proteins as well as genomic location and description of the primary transcript (as acquired from Phytozome Bd21 v3.1 database).

Assigned name of protein	Primary transcript ID (Phytozome)	Location	Description (Phytozome)
BdAGO1	Bradi2g10360.2	Bd2:8611187.8615652 reverse	PTHR22891//PTHR22891:SF44 – Eukaryotic translation initiation factor 2C
BdAGO2	Bradi2g14147.1	Bd2:12806099.12812784 reverse	PTHR22891:SF20 – Protein AGO 4-related
BdAGO3	Bradi2g10370.1	Bd2:8620394.8628745 reverse	AGO family, subfamily AGO4
BdAGO4	Bradi4g08587.1	Bd4:7715921.7724879 reverse	PTHR22891:SF35 – Protein AGO 6
BdAGO5	Bradi1g12431.2	Bd1:9307067.9313002 forward	PTHR22891//PTHR22891:SF49 – Eukaryotic translation initiation factor 2C
BdAGO6	Bradi1g05162.2	Bd1:3447373.3455769 forward	PTHR22891//PTHR22891:SF24 – Eukaryotic translation initiation factor 2C
BdAGO7	Bradi1g28260.3	Bd1:23482384.23489131 reverse	AGO family, subfamily monocot-AGO1
BdAGO8	Bradi1g16060.3	Bd1:12986117.12991032 reverse	AGO family, subfamily AGO7
BdAGO9	Bradi1g36907.2	Bd1:32760045.32772130 reverse	PTHR22891//PTHR22891:SF25 – Eukaryotic translation initiation factor 2C
BdAGO10	Bradi1g54977.1	Bd1:53536162.53543236 forward	PTHR22891//PTHR22891:SF36 – Eukaryotic translation initiation factor 2C
BdAGO11	Bradi1g29577.1	Bd1:25162908.25171156 reverse	PTHR22891//PTHR22891:SF57 – Eukaryotic translation initiation factor 2C
BdAGO12	Bradi5g18540.1	Bd5:21720455.21728815 reverse	AGO family, subfamily AGO1
BdAGO13	Bradi5g21810.1	Bd5:24487261.24492250 forward	AGO family, subfamily AGO2/3
BdAGO14	Bradi5g21800.1	Bd5:24479944.24484383 forward	AGO family, subfamily AGO2/3
BdAGO15	Bradi3g51077.3	Bd3:51944662.51956527 forward	PTHR22891//PTHR22891:SF34 – Eukaryotic translation initiation factor 2C
BdAGO16	Bradi3g60697.5	Bd3:59325332.59333596 reverse	PTHR22891//PTHR22891:SF34 – Eukaryotic translation initiation factor 2C

**Table 2:** Assigned names and accession numbers of BdDCL proteins as well as genomic location and description of the primary transcript (as acquired from Phytozome Bd21 v3.1 database).

Assigned name of protein	Primary transcript ID (Phytozome)	Location	Description (Phytozome)
BdDCL1	Bradi1g77087.1	Bd1:73701094.73713218 forward	PTHR14950:SF3 – ENDORIBONUCLEASE DICER HOMOLOG 1
BdDCL2a	Bradi1g15440.1	Bd1:12353426.12376799 forward	DCL family, subfamily DCL2
BdDCL2b	Bradi1g21030.3	Bd1:16934990.16948923 reverse	PTHR14950:SF19 - ENDORIBONUCLEASE DICER HOMOLOG 2
BdDCL3a	Bradi3g29287.1	Bd3:31008845.31020951 forward	PTHR14950//PTHR14950:SF31 - HELICASE-RELATED//SUBFAMILY NOT NAMED
BdDCL3b	Bradi2g23187.3	Bd2:20726122.20733365 reverse	PF00636//PF02170//PF03368 – Ribonuclease III domain (Ribonuclease_3)//PAZ domain (PAZ)//Dicer dimerization domain (Dicer_dimer)
BdDCL4	Bradi5g15337.3	Bd5:18845215.18867304 reverse	PTHR14950:SF15 – DCL 4

**Figure 1:**
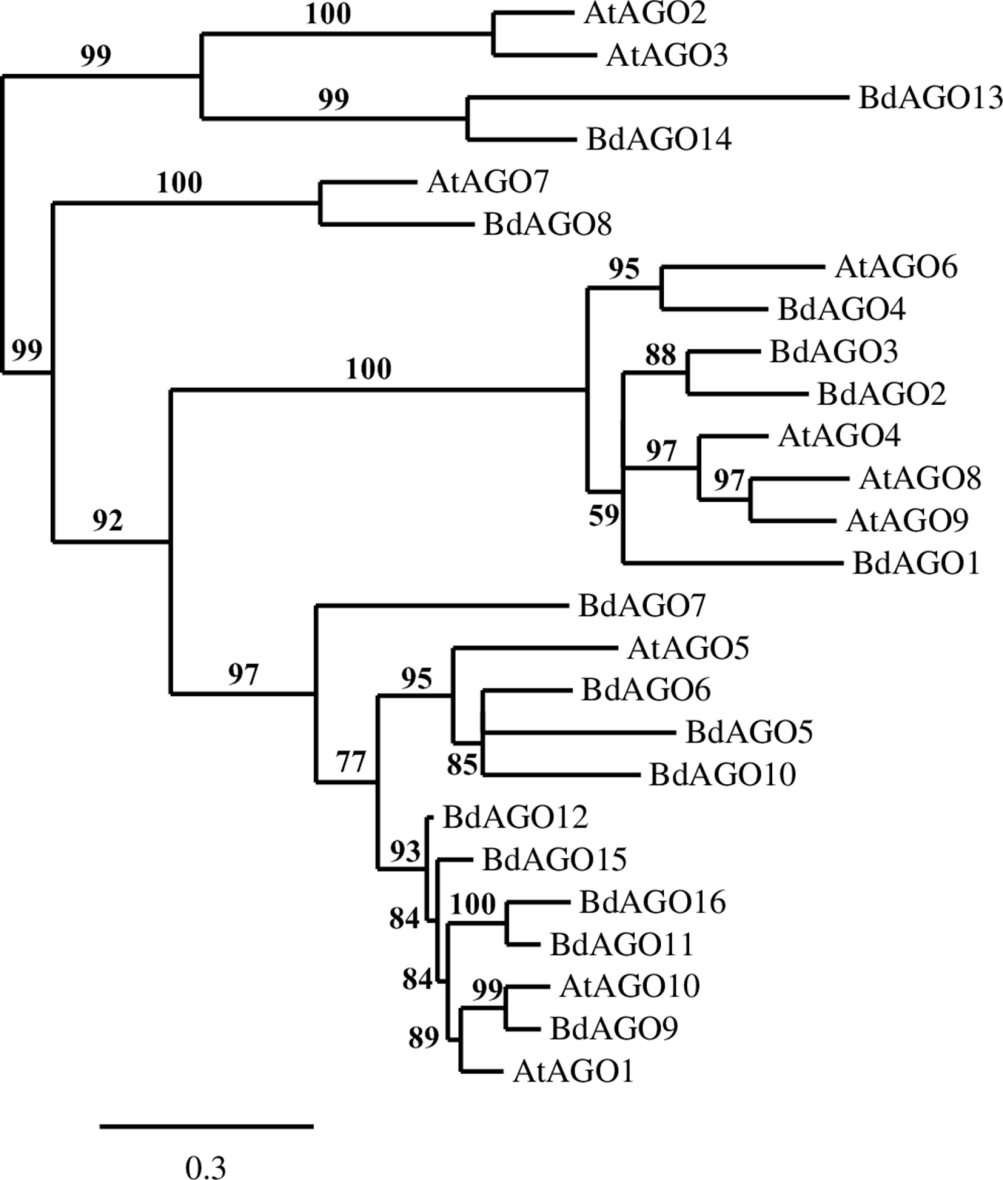
Phylogram of the BdAGO and AtAGO protein sequences, as calculated by Phylogeny.fr (MUSCLE, Gblocks, PhyML, TreeDyn). Branch support values are displayed in percentages, and branch support values smaller than 50% are collapsed. Scale bar defining the branch length displayed in bottom right corner.

**Figure 2:**
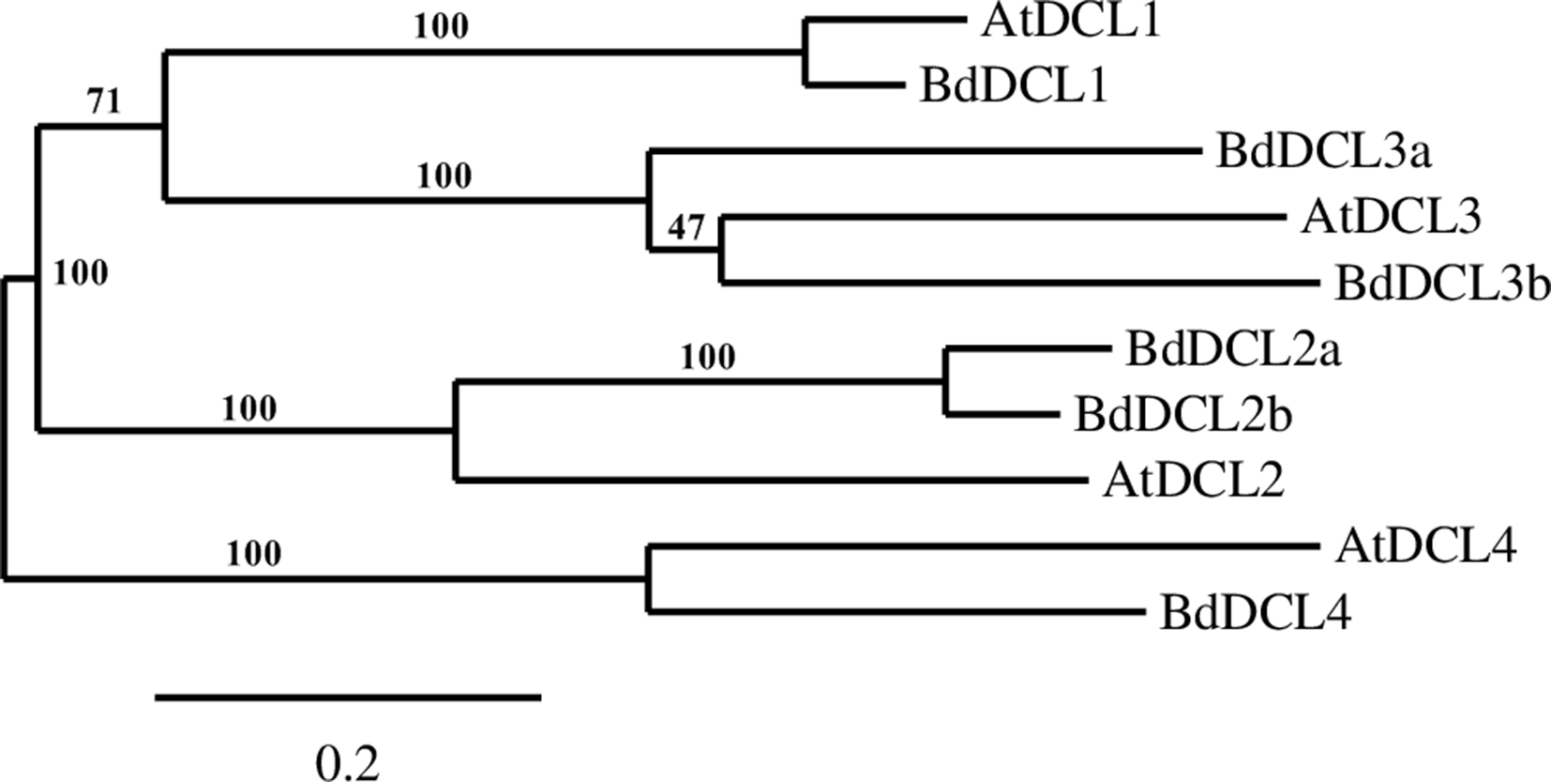
Phylogram of the BdDCL and AtDCL protein sequences, as calculated by Phylogeny.fr (MUSCLE, Gblocks, PhyML, TreeDyn). Branch support values are displayed in percentages, and branch support values smaller than 50% are collapsed. Scale bar defining the branch length displayed in bottom right corner.

A phylogenetic analysis of the inferred BdAGO protein sequences relative to those of *Arabidopsis* AGOs is shown in **Figure 1**. BdAGO proteins were placed in all three AtAGO clades. Some were grouped with a specific AtAGO member within a clade (e.g., BdAGO8 was grouped with AtAGO7, and BdAGO5/6/7/10 were grouped with AtAGO5), whereas other BdAGOs were distributed throughout an entire clade (e.g., BdAGO1/2/3/4 within the AtAGO4/6/8/9 clade). In the AtAGO1/5/10 clade, BdAGO9/11/12/15/16 were interspersed with AtAGO1 and AtAGO10. These findings suggest that the structural and functional differences of AtAGO proteins are translated to the expanded *Brachypodium* family. Phylogenetic analysis of the inferred BdDCL proteins showed that they strongly aligned with individual members of the *Arabidopsis* DCL family (**Figure 2**). Like *Arabidopsis*, *Brachypodium* contains a single ortholog of DCL1 and DCL4; however, expansion of the BdDCL family has led to the presence of two copies of both DCL2 and DCL3, as compared with the single ortholog present in *Arabidopsis*. Sequence comparisons revealed that DCL2a and DCL2b share 82.5% similarity at the aa level, while BdDCL3a and BdDCL3b share 44.2% similarity (aa, global alignment). Together, the phylogenetic trees show distinct branches interspersing *Arabidopsis* and *Brachypodium* homologues in functional clades; to our knowledge, this is the first indication of how the expansion of AGO and DCL protein families in *Brachypodium* relates to the specific clades and/or functional diversity of the corresponding *Arabidopsis* proteins.

### Predicted Domains of BdAGO and BdDCL Proteins Indicate Structure Conservation

**Figure 3:**
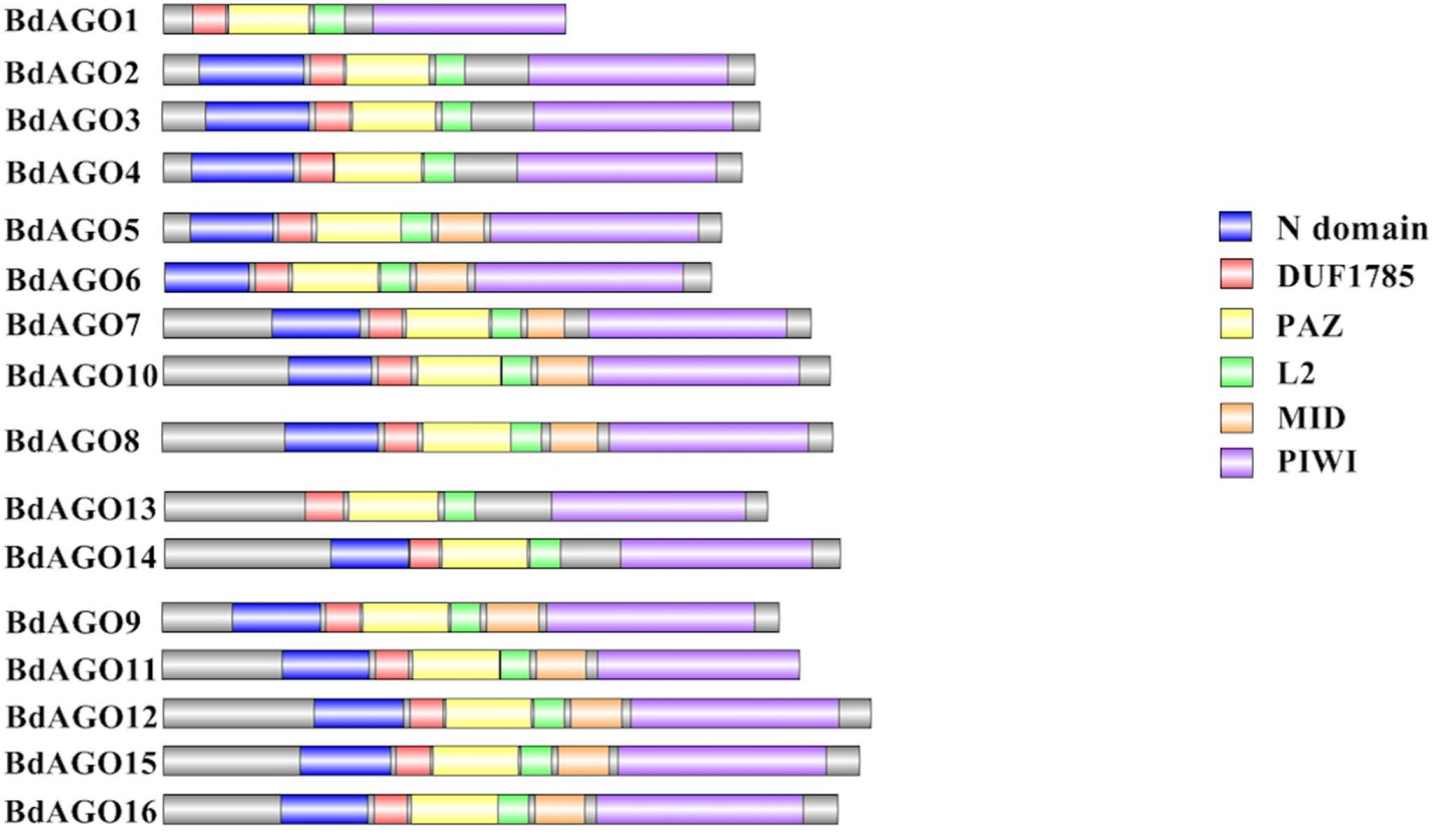
Visual representation of domain structure of BdAGO proteins, as identified by domain search by SMART and Pfam. Picture generated with Illustrator for Biological Sequences illustrator. Displayed domains: N-domain, DUF1785 (L1), PAZ (PIWI Argonaut and Zwille), L2, MID, PIWI, sequence, with no domain predicted in gray.

**Figure 4:**
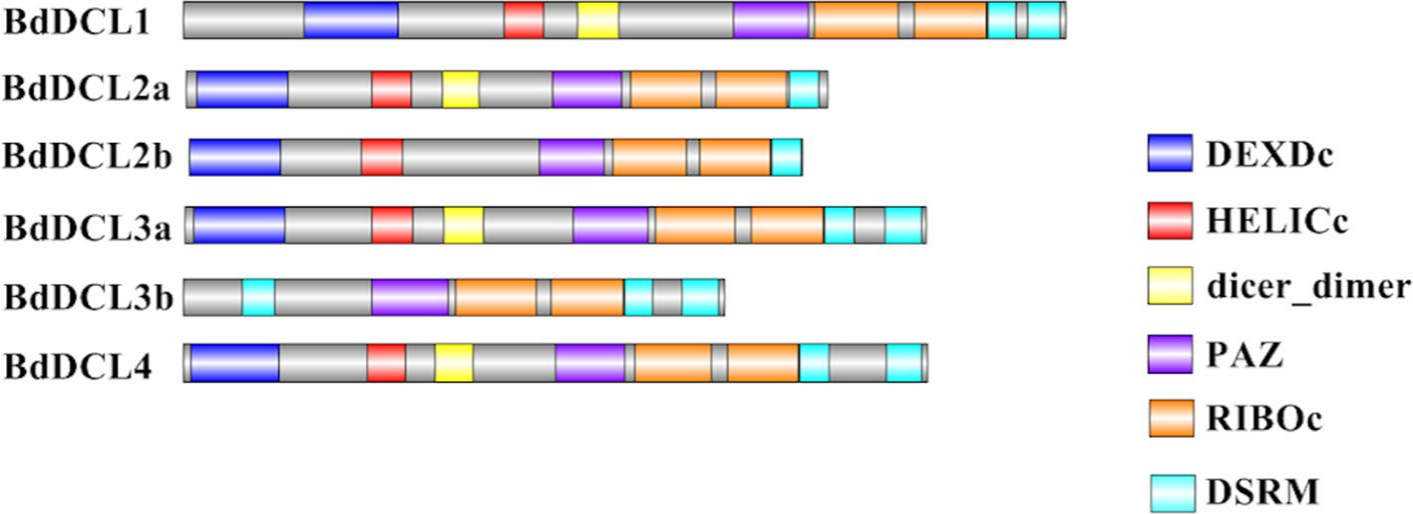
Visual representation of domain structure of Bd DCL proteins, as identified by domain search by SMART and Pfam. Picture generated with Illustrator for Biological Sequences illustrator. Displayed domains: DEAD-like helicase superfamily (DEXDc), helicase superfamily c-terminal domain (HELICc), dicer_dimer, PIWI Argonaut and Zwille (PAZ), ribonuclease III family (RIBOc), double-stranded RNA-binding motif (DSRM), sequence with no domain predicted in gray.

**Figure 5:**
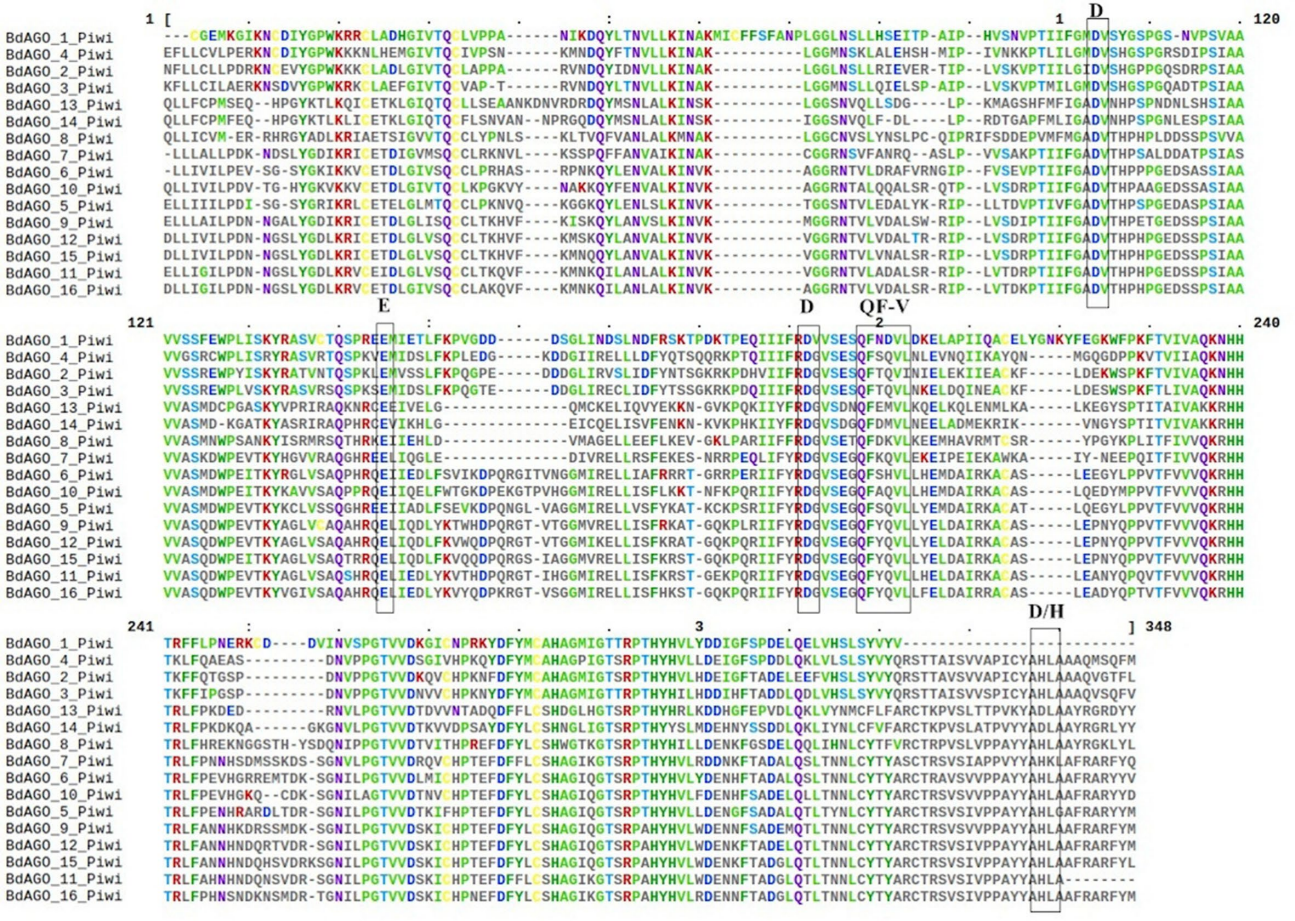
Multiple sequence alignment (MSA) of the PIWI domain of BdAGO proteins, as acquired by Clustal Omega and Mview visualization. The catalytic tetrad DEDD/H (Asp-Glu-Asp-Asp or Asp-Glu-Asp-His) and the QF-V (Gln-Phe-Val) motifs are boxed.

Next, we executed a domain search using SMART to elucidate the structures and functions of the 16 BdAGO proteins and six BdDCLs. The domain structure visualization of BdAGO (**Figure 3**) and BdDCL (**Figure 4**) proteins highlights the differences between members of each protein family with respect to the positions and presence/absence of the typically conserved domains. Detailed domain prediction data, as acquired by SMART/Pfam search, and the corresponding confidence values are shown for BdAGO (**Table S2**) and BdDCL proteins (**Table S3**). Consistent with other eukaryotic AGO proteins, many members of the BdAGO family are predicted to have four characteristic functional domains, including the N-terminal domain, PAZ, MID, and PIWI domain (Zhang et al., 2014). However, while the domain prediction results identified a variable N-t domain in most BdAGOs that consisted of both an N-domain and a DUF1785 domain (Poulsen et al., 2013), BdAGO1 and BdAGO13 contained only the DUF1785 domain. In addition, BdAGO1, BdAGO2, BdAGO3, BdAGO4, BdAGO13, and BdAGO14 were not predicted to contain a MID domain, in comparison to a previous report (Mirzaei et al., 2014). MSA performed by Clustal Omega on the PIWI domain of BdAGO proteins (**Figure 5**) showed a typical pattern of conservation for the DEDD/H catalytic tetrad required for slicer activity and a conserved QF-V motif in all aligned sequences except BdAGO1, which has the shortest protein sequence of all the BdAGO proteins and lacks the D/H residue of the catalytic tetrad.

Analysis of the predicted domains in BdDCL proteins revealed that the characteristic DEXDc, HELICc, Dicer-dimer (DUF283, Qin et al., 2010), PAZ, RIBOc, and DSRM domains are present in most family members. However, BdDCL2b and BdDCL3b lack the dimerization domain; BdDCL3b additionally lacks both the DEXDc and HELICc domains and instead contains an additional DSRM domain at the N terminus (position: 131-218, E-value: 8.6e-17; **Figure 4** and **Table S3**). By contrast, BdDCL2a and BdDCL2b contain only one DSRM domain (**Figure 4**, **Table S3**).

### Three-Dimensional Modeling Supports Phylogenetic Data Showing a Strong Expansion in the BdAGOs in the AGO1/5/10-Related Clade

**Figure 6:**
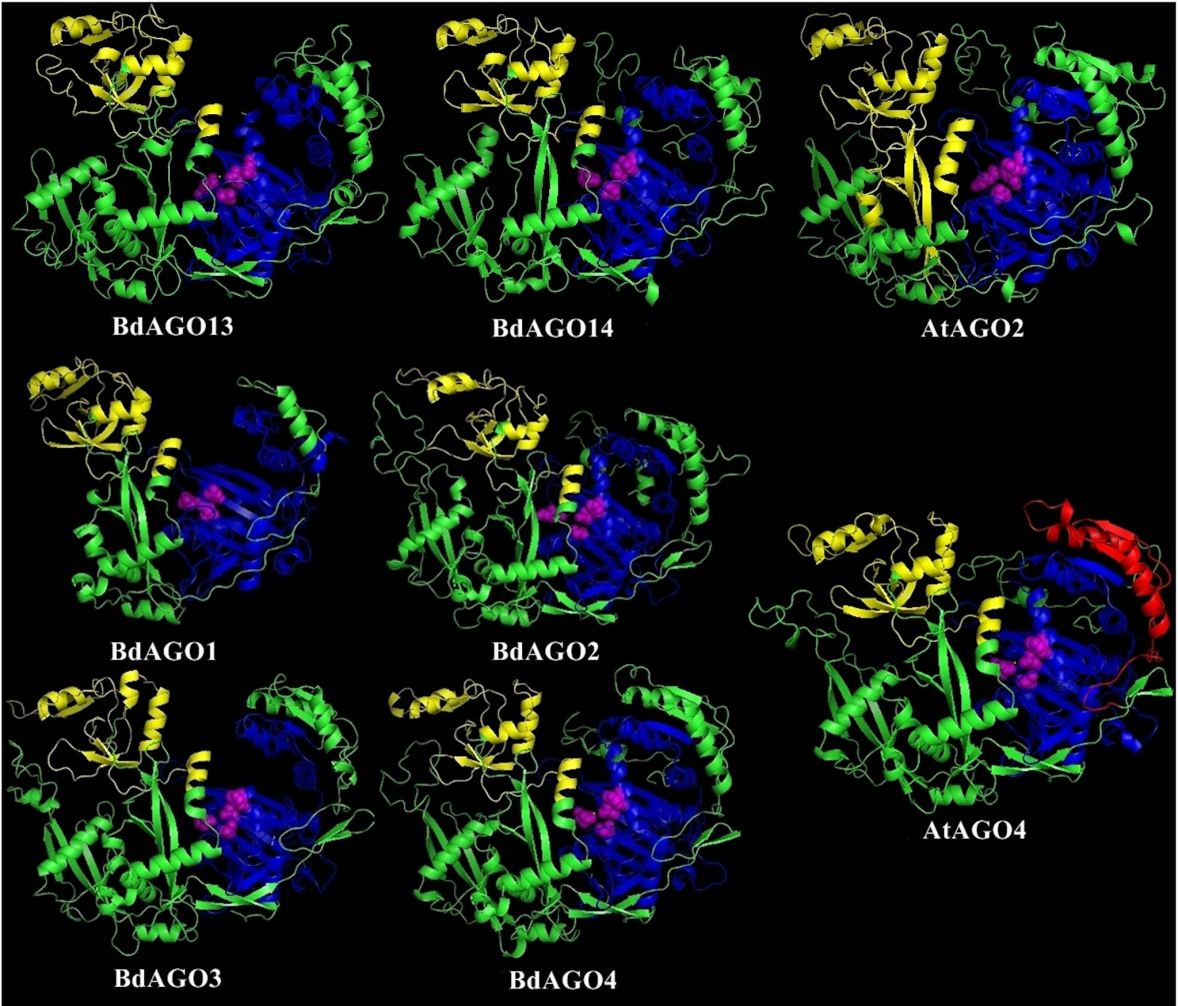
Three-dimensional structure predictions for BdAGO13 and BdAGO14 (with AtAGO2 as the closest homolog in *Arabidopsis*) and BdAGO1, BdAGO2, BdAGO3, BdAGO4 (with AtAGO4 as the closest homolog in *Arabidopsis*), as modeled by SWISS-MODEL. PAZ (yellow), PIWI (blue), and MID (red) domains as predicted by SMART and Pfam displayed. The catalytic tetrad within the PIWI domain (DEDD) is marked by magenta spheres. Visualization by PyMOL.

**Figure 7:**
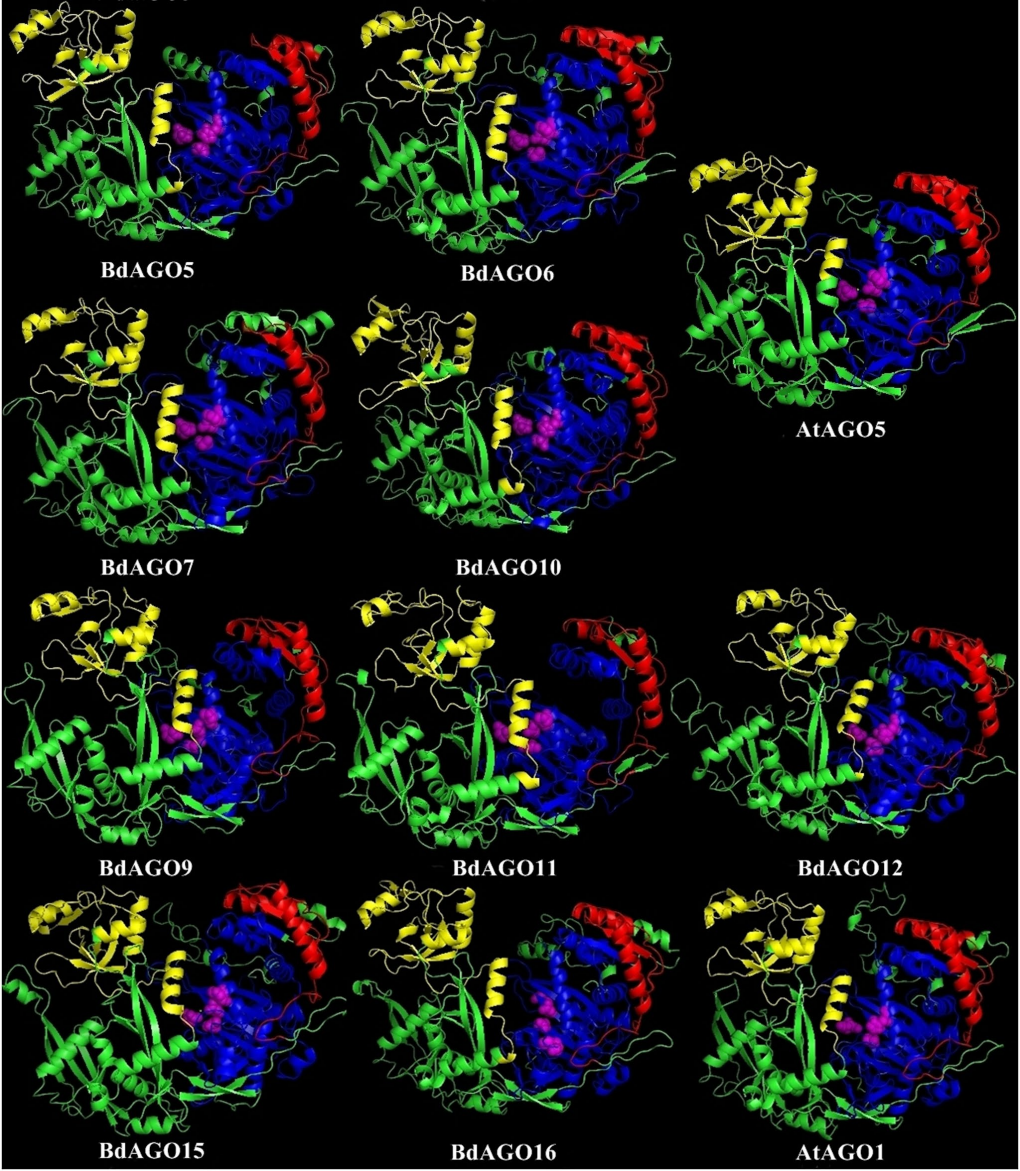
Three-dimensional structure predictions for BdAGO5, BdAGO6, BdAGO7, and BdAGO10 (with AtAGO5 as the closest homolog in *Arabidopsis*) and BdAGO9, BdAGO11, BdAGO12, BdAGO15, and BdAGO16 (with AtAGO1 as the closest homolog in *Arabidopsis*), as modeled by SWISS-MODEL. PAZ (yellow), PIWI (blue), and MID (red) domains as predicted by SMART and Pfam displayed. The catalytic tetrad within the PIWI domain (DEDD) is marked by magenta spheres. Visualization by PyMOL.

In order to obtain an optimal homology-based 3D model of the studied proteins, we used SWISS-MODEL and CPHmodels 3.2. When choosing between models generated by alternative software programs or based on different templates, validation of the predicted structures is crucial for generating a consensus on the optimal model and further comparison. In case of BdAGOs, validation of the predicted structures was done using four different measurements, the results of which are shown in **Table S4**. While a 0-1 QMEAN value gives an absolute scoring of the predicted model, the Z-score shown in **Table S4** serves as a comparison of the quality of the prediction of the query model relative to expected from a high-resolution X-ray crystallography structure. Typically, the more negative the Z-score is, the lower the quality of the predicted structure. Using PROCHECK, we report on the percentage of residues that fall into the most favored regions of the Ramachandran plot. The free energy score of the conformation of the predicted protein calculated by dDFIRE usually indicates lower values for a better model. Based on validation of the 3D models by the software, SWISS-MODEL was chosen as the preferred modeling tool for BdAGO proteins (**Table S4**). The corresponding AtAGO 3D structures, predicted and validated in the same fashion (**Table S5**), were subsequently used alongside the visualization of the BdAGO proteins by PyMOL. **Figures 6**, **7**, and **S1** display the models, in which the PAZ, MID, and PIWI domains (where predicted) and residues comprising the DEDD/H catalytic tetrad are indicated for all BdAGOs and a corresponding AtAGO representing the appropriate branch of the phylogenetic tree depicted in **Figure 1**. Overall, the predicted structures of the BdAGOs mirror the corresponding AtAGO structures, suggesting a functional conservation. The PIWI domain and the catalytic tetrad especially show similarity between the clade members shown together in **Figures 6** and **7**. The BdDCL proteins did not have successfully modeled structures predicted by either software and thus are not shown.

### Expression Analysis and Putative Interactors of BdAGO Proteins

We addressed the question of tissue-specific expression of *BdAGO* and *BdDCL* genes by utilizing the *B. distachyon* eFP Browser (Sibout et al., 2017; Winter et al., 2007) in **Table 3**. Stronger expression of *BdAGO* and *BdDCL* genes was observed in seed and stem tissue compared with roots or leaves. The plant subcellular localization integrative predictor used for protein localization predicted that all BdAGOs reside in the cytosol, except for BdAGO3, BdAGO14 (predicted to localize in the nucleus), and BdAGO7 (predicted to localize in plastids), with varying scores of confidence (**Table S7**).

**Table 3:** Gene expression data as displayed on the *B. distachyon* eFP Browser (Winter et al., 2007; Sibout et al., 2017).

Gene ID	Assigned name	^1^Highest expression signal	^2^Peduncle, spikelet and stem nodes	^2^Root	^2^Leaf	^2^Seed
Bradi2g10360	BdAGO1	Whole_grain_11_DAF				
Bradi2g14147	BdAGO2	First_node_27_DAG				
Bradi2g10370	BdAGO3	First_node_10_DAG				
Bradi4g08587	BdAGO4	Whole_grain_2_years				
Bradi1g12431	BdAGO5	Not found in browser				
Bradi1g05162	BdAGO6	Endosperm_31_DAF				
Bradi1g28260	BdAGO7	Endosperm_11_DAF				
Bradi1g16060	BdAGO8	First_node_10_DAG				
Bradi1g36907	BdAGO9	First_node_10_DAG				
Bradi1g54977	BdAGO10	Whole_grain_11_DAF				
Bradi1g29577	BdAGO11	Upper_part_of_inclined_node_42_DAG				
Bradi5g18540	BdAGO12	Last_internode_35_DAG				
Bradi5g21810	BdAGO13	Last_node_35_DAG				
Bradi5g21800	BdAGO14	Last_internode_35_DAG				
Bradi3g51077	BdAGO15	Roots_10_DAG				
Bradi3g60697	BdAGO16	Roots_10_DAG				
Bradi1g77087	BdDCL1	Whole_grain_2_years				
Bradi1g15440	BdDCL2a	First_internode_27_DAG				
Bradi1g21030	BdDCL2b	Whole_grain_2_years				
Bradi3g29287	BdDCL3a	Lower_part_of_inclined_node_42_DAG				
Bradi2g23187	BdDCL3b	First_node_10_DAG				
Bradi5g15337	BdDCL4	Whole_grain_2_years				

^1^The tissue with the highest absolute expression level per gene ID is indicated (DAF, day after fertilization; DAG, day after germination).

^2^Summary of relative expression level per gene ID displayed for tissues; color indicates relative expression levels (log2, the control is calculated from all the samples displayed on the particular eFP browser view); dark blue (high for the transcript relative to control) to light blue (low for the transcript relative to control).

Finally, prediction of proteins that interact with BdAGOs was carried out using STRING (**Table S6**). All predicted BdAGOs were found to be either co-expressed or experimentally shown to interact with three proteins: Bradi1g36340.1, Bradi2g30160.1, and Bradi4g45065.1. BLASTP search of these protein sequences identified them as a 110-kDa U5 small nuclear ribonucleoprotein component CLO (Bradi1g36340.1), a putative GTP-binding/transcription factor (Bradi2g30160.1), and DNA-directed RNA polymerase V subunit 1 or DNA-directed RNA polymerase V subunit 1 (Bradi4g45065.1). In addition to the aforementioned proteins, BdAGO9 (classified in the AtAGO1/5/10 clade) was predicted to interact with seven other proteins, identified as three homeobox proteins knotted-1-like (Bradi1g12677.1, Bradi1g12690.1, Bradi1g57607.1), two GATA transcription factors (Bradi2g14890.1, Bradi2g45750.1), and two putative uncharacterized proteins (**Figure S2**).

## Discussion

In the present work, we investigated the phylogenetic relationships, domain, structure conservation, and predicted redundancy of AGO and DCL proteins in the model grass plant *B. distachyon*. Our findings imply that BdAGOs and BdDCLs have more copies and possibly greater diversification relative to *Arabidopsis*. One known example of such diversification in monocotyledonous plants is the rice AGO18, which confers antiviral immunity by sequestration of an miRNA (Wu et al., 2015). Since the presence of domains typical for AGO and DCL protein families serves as a selection criterion for proteins within this uninvestigated grass model species, we discuss phylogenetic relationships and predicted domain occurrence in detail.

Our analyses show that *Brachypodium*, like other grasses, contains one protein (BdDCL1) whose sequence groups with AtDCL1, one with AtDCL4 (BdDCL4), and two proteins each that group with AtDCL2 and AtDCL3 (Margis et al., 2006). Analysis of their predicted domain structures showed that BdDCL2b and BdDCL3b lack the dicer-dimer (DUF283) domain, known to mediate heterodimerization of AtDCL4 with its protein partners (Qin et al., 2010), but it is partially missing in two other DCLs (AtDCL3 and OsDCL2b, Margis et al., 2006). The second DSRM domain also was not predicted in either of the BdDCL2s. This finding is consistent with the previous discovery that AtDCL2 in *Arabidopsis* and OsDCL2a and OsDCL2b in rice also contain only one DSRM (Margis et al., 2006). This second DSRM domain has only a weak affinity for dsRNA, but it specifically binds to proteins of the HYPONASTIC LEAVES 1/dsRNA-binding protein family (Hiraguri et al., 2005; Margis et al., 2006). Since the DSRM domains mediate the transfer of the newly generated sRNA to the appropriate AGO protein (Parker et al., 2008), variations in the C-terminal architecture may influence which downstream partners and RNAi pathways are utilized by specific DCLs. The high level of divergence between DCL3a and DCL3b in several monocot species has led to the classification of DCL3b as a distinct type of DCL, termed DCL5. This monocot-specific class of DCLs has been retained for over 60 million years (Margis et al., 2006). It is is responsible for generating 24-nt-phased sRNAs in the male reproductive organs (Song et al., 2012). Interestingly, the predicted domain structure of BdDCL3b differs substantially from that of BdDCL3a, as it lacks both the DEXDc and HELICc domains (alongside the dicer-dimer domain) but contains an additional N-terminal DSRM (**Figure 4**, **Table S3**). Since the helicase domains are thought to mediate unwinding of the dsRNA (Zhang et al., 2004), the functionality of BdDCL3b is unclear. Mutations in AtDCL1 that impair helicase activity were previously shown to suppress miRNA accumulation (Liu et al., 2012). However, comparable levels of transcripts for two splice variants of AtDCL2, one of which contains an altered helicase region, were detected throughout the *Arabidopsis* life cycle (Margis et al., 2006). Additional structural and biochemical analyses are therefore required to assess the role of BdDCL3b.

Phylogenetic analysis of the BdAGO protein family placed members in all three clades defined by *Arabidopsis* AGOs. Thus, the structural and functional differences of AtAGO proteins appear to be translated to the expanded *Brachypodium* family. For two of the three clades, the number of BdAGO and AtAGO family members was equivalent. By contrast, the AtAGO1/5/10 clade was highly expanded in *Brachypodium*, with four members (BdAGO9/11/12/15/16) grouped with AtAGO1. This member of the *Arabidopsis* family is associated with a range of functions, including processing of dsRNA from transgenes and exogenous sources, and RNAs involved in ckRNAi (Vaucheret et al., 2004; Weiberg et al., 2013). If the corresponding BdAGO members of this clade are found to have similar functions in PTGS-mediated transgene silencing, this information would be highly useful for developing RNAi-based protection strategies for cereal crops. AtAGO10 groups with the same BdAGOs, as expected considering the clade association with AtAGO1. AtAGO5, which is the third member of the AtAGO1/5/10 clade, groups with BdAGO5/6/7/10. By contrast, BdAGO1/2/3/4 were interspersed within the AtAGO4/6/8/9 clade, raising the possibility that these *Brachypodium* proteins are involved in TGS.

As displayed in our domain visualization (**Figure 3**), all 16 BdAGOs have a predicted PAZ domain. In AGOs, this domain recognizes the 3’ end of the guide sRNA molecule, made accessible to the hydrophobic pocket of this nucleotide-binding domain by the typical 2’-O-methyl modification of the final sugar (Lingel et al., 2003; Cenik and Zamore, 2011). The MID domain recognizes the 5’ nucleotide of the sRNA, thus giving preference of an AGO protein into which the sRNA will be loaded (Frank et al., 2012). In *Arabidopsis*, sRNA with a 5’ U are sorted into AtAGO1, while AtAGO2 and AtAGO4 load sRNAs with a 5’ A and AtAGO5 loads sRNAs with a 5’ C (Mi et al., 2008). Our SMART/Pfam domain architecture search failed to identify a MID domain in any of the BdAGOs grouped in the AtAGO4/6/8/9 clade (BdAGO1/2/3/4) and with the AtAGO2/3 (BdAGO13/14) (**Table S2**), although this domain was reported in these proteins in a different study (Mirzaei et al., 2014). The specificity of sRNA sorting into particular AGOs can be further determined by the recognition of the sRNA secondary structure/base pairing by a QF-V motif present in the PIWI domain (Zhang et al., 2014). All *Arabidopsis* AGOs have the conserved QF-V motif, as do all 16 BdAGOs (**Figure 5**). The DEDD/H catalytic tetrad in the PIWI domain is also present in all but one of the BdAGOs. These active-site residues are critical for the RNase H-like endonuclease (slicer) activity exhibited by certain AGOs, which mediates sequence-specific cleavage of the target transcript. AtAGO1, AtAGO2, AtAGO4, AtAGO7, and AtAGO10 have been shown to have endonucleolytic activity toward target RNAs (Fang and Qi, 2016). Originally identified as a catalytic triad consisting of the residues DDH in most AtAGOs, but DDD in AtAGO2 and AtAGO3 (Höck and Meister, 2008), studies of yeast AGO revealed the importance of an invariant glutamate (E) residue, creating a catalytic tetrad (Nakanishi et al., 2012). This E residue is conserved in all *Arabidopsis* AGOs (Zhang et al., 2014). Consistent with these findings, MSA visualization of the BdAGO PIWI domains indicated that the majority of BdAGO proteins have the DEDH tetrad, except BdAGO13 and BdAGO14, which like their closest homologues AtAGO2 and AtAGO3, contain the DEDD tetrad (**Figure 5**). The only exception is BdAGO1, which is a short protein that terminates after 624 residues and lacks the last catalytic residue of the tetrad. Without the conserved catalytic residues, a specific AGO protein might induce gene silencing through means other than cutting, but Höck and Meister (2008) also discuss that the presence of a conserved catalytic triad does not mean the protein indeed has endonuclease activity. If an AGO does not display endonuclease activity, it may mediate PTGS *via* translation inhibition of the target RNA (Carthew and Sontheimer, 2009). Interestingly, the L1 and L2 linkers are predicted in all 16 BdAGOs as well (**Table S2**).

3D structure visualizations of all BdAGOs (**Figures 6**, **7** and **S1**) reinforce the conservation of the PAZ, PIWI, and MID domains (when predicted by SMART) and the catalytic tetrad residues in proximity within the PIWI domain (magenta spheres). The differences in the folding and looping linker regions within a certain group, relative to *Arabidopsis* AGOs, are shown in the model visualizations. Furthermore, the similarity between the 3D structures of BdAGOs that were predicted either to contain or lack a MID domain by the SMART/Pfam domain architecture search (e.g., **Figure 6**) reinforces the importance of comparing entire 3D models in order to gain insight into structure/function conservation. These structures are based on templates with better-known functional specificity and thus hint at the functions of the orthologs in Bd. As shown in **Table S4**, the templates used for modeling are based on either Argonaute 1 or Argonaute 2, with varying coverage and confidence values.

To assess the expression levels and locations of BdAGO and BdDCL family members, we analyzed the microarray-based expression data in the *B. distachyon* eFP browser (**Table 3**). Expression of *BdAGO* genes was observed in all four tissues assayed, although to varying extents. The expression patterns across the gene families indicate potential for functional redundancy. Notably, all members of the AtAGO1 clade (*BdAGO9/11/12/15/16*) show high and intermediate levels of expression in stem nodes and root tissue, respectively, while the *BdAGO1/2/3/4* proteins generally display high expression in stem nodes and seeds. Analysis of *BdDCL* gene expression revealed that most members of this family are highly expressed in stem nodes and/or seeds. *In vivo* experimental approaches are necessary to decipher whether the apparent co-expression of these genes indicates specific compartmentalization or complete/partial redundancy in the various RNAi processes, including environmental RNAi and ckRNAi pathways.

Finally, we used STRING to predict the interactome for members of the BdAGO family. This analysis indicates that all BdAGOs interact with three proteins (**Table S6**), as was expected because of the domain conservation within the family. In addition, several potential interactors were identified for BdAGO9, based on co-expression or experimental data (**Figure S2**, **Table S6**). Of these, DNA-directed RNA polymerase V subunit 1 was previously shown to co-localize with, and possibly directly bind to, AtAGO4 *via* a so-called “Ago hook” (GW-rich domain), in order to facilitate the recruitment of AGO4 to chromatin to mediate TGS (El-Shami et al., 2007; Fang and Qi, 2016). Poulsen et al. (2013) have discussed that the binding of GW interactors to AGO make the loop with the E residue of the catalytic tetrad unavailable to the otherwise rigid DDD/H triad within the PIWI domain, thus offering an explanation of how the slicing activity is prevented in cases of silencing by translational inhibition. Moreover, GW containing proteins Needed for RDR2-independent DNA methylation and Silencing Defective 3 have been indicated in pathways bringing DNA/chromatin silencing together with RNAi proteins (Garcia et al., 2012; Pontier et al., 2012). Protein co-expression and interaction studies *in vivo* are necessary to confirm the identity and locations of these putative BdAGO interacting proteins. Due to the stringency of the prediction (excluding text mining data) and the lack of knowledge about the *Brachypodium* RNAi machinery, we were unable to predict additional interactions or to detect RNAi-related proteins that are known to interact with members of the AGO family in *Arabidopsis*. These include DCLs, HEN1 (involved in the methylation of sRNA 3’ ends to prevent degradation), RDRs (RNA-dependent RNA polymerases that synthesize dsRNAs from single-stranded RNAs), and HSP90, the heat shock protein that binds to AtAGO1 and AtAGO4 to aid the loading of the sRNAs and RISC assembly (Fang and Qi, 2016; Nakanishi, 2016). Moreover, the predicted localization of the BdAGOs places the majority of them in the cytosol, except for BdAGO3 and BdAGO14, which are predicted to localize in the nucleus (**Table S7**). From what is known about *Arabidopsis* AGOs, AtAGO1 is proposed to have a localization in the nucleus and the cytoplasm (Vaucheret, 2008), while AtAGO4 localizes to the nuclear Cajal bodies (Höck and Meister, 2008).

In sum, based on *in silico* prediction, our data provide the first detailed functional insight into the AGO and DCL protein families in *Brachypodium*. In the context of plant–microbe interactions and ckRNAi, the *Brachypodium* orthologs of AtAGO1 are of special interest because microbial sRNAs are shown to be loaded onto AtAGO1 (Weiberg et al., 2013). Our predictions indicate a clade of BdAGOs structurally similar to AtAGO1, consisting of BdAGO9/11/12/15/16 (**Figure 7**). Elaborating on such similarities with the well-established clades of *Arabidopsis* AGOs and DCLs is a valuable basis for testing the hypothesis that BdAGO9/11/12/15/16 proteins are required for exogenous and endogenous dsRNA processing in HIGS, SIGS, and bidirectional ckRNAi in the grass model. Beyond what is predictable by *in silico* analysis, more data on expression patterns and interacting proteins are needed to further understand the role of these pillar proteins of RNAi pathways in cereals.

## Data Availability Statement

All datasets for this study are included in the article/**Supplementary Files**.

## Author Contributions

EŠ, SZ, and KHK wrote the text. EŠ and SZ performed analysis and designed the figures.

## Funding

This work was funded by the Deutsche Forschungsgemeinschaft in the program GRK2355 to KHK and by of the European Union in the Marie Skłodowska-Curie Actions CEREALPATH to KHK and SZ.

## Conflict of Interest

The authors declare that the research was conducted in the absence of any commercial or financial relationships that could be construed as a potential conflict of interest.
