# Supplementary material for: Further Elucidation of the Argonaute and Dicer Protein Families in the Model Grass Species Brachypodium distachyon
Source: Front Plant Sci. 2019 Oct 22;10:1332. doi: 10.3389/fpls.2019.01332 (PMC6822278; doi:10.3389/fpls.2019.01332)

Supplementary Material

# Supplementary Tables

**Table S1: Accession numbers of *AtAGO* and *AtDCL* gene IDs, as acquired from TAIR.**

| **Assigned name of protein** | **Gene ID (TAIR)** |
| --- | --- |
| AtAGO1 | AT1G48410.2 |
| AtAGO2 | AT1G31280.1 |
| AtAGO3 | AT1G31290.1 |
| AtAGO4 | AT2G27040.1 |
| AtAGO5 | AT2G27880.1 |
| AtAGO6 | AT2G32940.1 |
| AtAGO7 | AT1G69440.1 |
| AtAGO8 | AT5G21030.1 |
| AtAGO9 | AT5G21150.1 |
| AtAGO10 | AT5G43810.1 |
| AtDCL1 | AT1G01040.1 |
| AtDCL2 | AT3G03300.1 |
| AtDCL3 | AT3G43920.2 |
| AtDCL4 | AT5G20320.1 |

**Table S2: Domain structure of Bd AGO-like proteins, as identified by domain search by SMART, with PFAM overlaps of higher confidence shown in some cases. Domains are indicated with a start-end residue interval, followed by E-values. Domains shown: N domain, Linker 1 (DUF1785), PAZ, Linker 2 (L2), MID and PIWI.**

| **Protein** | **N domain** | **DUF1785** | **PAZ** | **L2** | **MID** | **PIWI** |
| --- | --- | --- | --- | --- | --- | --- |
| BdAGO1 |  | 46-97 (1.34e-17) | 101-225 (1.1e-23) | 234-281 (4.4e-13) |  | 325-623 (2.02e-56) |
| BdAGO2 | 56-218 (3.3e-34) | 228-280 (2.37e-18) | 284-412 ( 3e-26) | 421-467 (4.8e-14) |  | 565-874 (8.55e-110) |
| BdAGO3 | 68-228 ( 8.8e-34) | 238-290 (7.82e-19) | 294-424 (1.3e-27) | 433-479 (6e-16) |  | 576-883 ( 5.43e-112) |
| BdAGO4 | 38-196 (2.3e-31) | 206-258 (2.27e-15) | 262-389 (2.4e-25) | 398-444 (2.1e-16) |  | 541-850 (1.21e-109) |
| BdAGO5 | 42-170 (8.4e-35) | 179-230 (8.4e-35) | 237-368 (0.000638) | 369-415 (1.6e-17) | 425-496 (3.9e-11) | 507-827 (5.21e-123) |
| BdAGO6 | 1-132 (5.9e-32) | 141-193 ( 1.6e-24) | 198-330 (0.00339) | 334-380 (1e-13) | 390-469 (1.1e-11) | 481-803 (5.01e-125) |
| BdAGO7 | 169-304 (7.1e-31) | 318-370 (9.7e-24) | 375-504 (0.00000249) | 508-554 (1.2e-14) | 564-621 (6.6e-9) | 658-964 (5.77e-116) |
| BdAGO8 | 191-335 (3.5e-24) | 344-396 (4.07e-17) | 404-544 (0.000953) | 539-586 ( 1.1e-7) | 601-673 ( 0.0077) | 692-1000 (5.03e-107) |
| BdAGO9 | 110-245 (2.1e-34) | 254-306 (1.1e-22) | 311-443 (0.00232) | 447-493 (9e-18) | 503-583 ( 6e-15) | 595-916 (7.09e-125) |
| BdAGO10 | 195-323 (2.6e-33) | 332-383 (3.38e-23) | 394-522 (0.00188) | 523-569 (3.4e-17) | 579-658 (4.1e-12) | 664-983 (2.75e-122) |
| BdAGO11 | 186-321 (9.7e-35) | 330-382 (1.18e-22) | 387-522 (0.0000324) | 523-569 (3.2e-15) | 579-656 (5.4e-9) | 673-986 (1.48e-113) |
| BdAGO12 | 233-372 (5.4e-37) | 381-433 (2.11e-24) | 438-570 (0.00184) | 574-620 (1.1e-18) | 630-710 (2e-11) | 724-1045 (1.22e-128) |
| BdAGO13 |  | 218-278 (0.000311) | 286-423 (2.7e-18) | 434-481 (2.8e-13) |  | 599-899 ( 3.56e-98) |
| BdAGO14 | 251-373 (7.5e-14) | 374-419 (0.000167) | 423-550(3.8e-23) | 560-607 (1.6e-10) |  | 699-996 (1.6e-10) |
| BdAGO15 | 213-352 (2.7e-36) | 361-413 (1.28e-23) | 418-550 ( 0.00155) | 554-600 ( 1.1e-17) | 610-689 ( 1.3e-10) | 704-1026 (1.36e-126) |
| BdAGO16 | 183-317 (3.7e-37) | 326-378 ( 9.7e-24) | 383-518 (0.000464) | 519-565 ( 8.5e-18) | 575-652 ( 4.5e-10) | 669-990 ( 7.9e-123) |

**Table S3: Domain structure of BdDCLs, as identified by domain search by SMART, with PFAM overlaps of higher confidence shown in some cases. Domains are indicated with a start-end residue interval, followed by E-values. Domains shown: DEXDc, HELICc, Dicer_dimer, PAZ, RIBOc, DSRM.**

| **Protein** | **DEXDc** | **HELICc** | **Dicer_dimer** | **PAZ** | **RIBOc** | **RIBOc** | **DSRM** | **DSRM** |
| --- | --- | --- | --- | --- | --- | --- | --- | --- |
| BdDCL1 | 266-469 (9.68e-20) | 703-789 (5.28e-12) | 862-952 (6.6e-24) | 1203-1366 (1.43e-29) | 1381-1564 (6.27e-34) | 1600-1756 (5.62e-45) | 1760-1821 (1.53e-6) | 1845-1918 (2.15e-10) |
| BdDCL2a | 23-223 (3.78e-22) | 406-493 (1.35e-18) | 561-642 (1.6e-18) | 810-952 (2.08e-8) | 972-1127 (1.12e-21) | 1159-1314 (5.06e-29) | 1318-1382 (0.0221) |  |
| BdDCL2b | 1-201 (3.93e-20) | 379-466 (1.54e-13) |  | 766-908 (0.0000604) | 928-1086 (2.76e-21) | 1116-1271 (9.08e-31) | 1275-1339 (0.000000573) |  |
| BdDCL3a | 22-219 (4.62e-17) | 410-500 (3.76e-17) | 567-653 (1.3e-26) | 849-1014 (0.000611) | 1031-1204 (2.65e-23) | 1240-1396 (2.51e-33) | 1400-1463 (0.0000011) | 1530-1612 (0.423) |
| BdDCL3b |  |  |  | 434-577 (6.8e-15) | 596-772 (6.73e-25) | 805-961 (2.22e-34) | 965-1028 (0.201) | 1090-1172 (0.806) |
| BdDCL4 | 17-211 (2e-21) | 404-486 (2.64e-12) | 550-636 (8.5e-24) | 813-967 (5.81e-7) | 988-1156 (9.04e-17) | 1192-1344 (2.63e-27) | 1348-1413 (0.912) | 1539-1613 (9.59e-8) |

**Table S4: Validation of predicted 3D structures of BdAGO-like proteins. Prediction with CPH and Swiss Model, validation by QMEAN, PROCHECK, WHATCHECK and dFIRE. Last column indicates the template used for the model and coverage.**

| **Protein** | **3D Modelling software** | **QMEAN (Z-score)** | **Residues in favored region (PROCHECK)** | **Ramachandran z-score (WHATCHECK)** | **dDFIRE total energy** | **Model used (coverage)** |
| --- | --- | --- | --- | --- | --- | --- |
| BdAGO1 | CPH | -6.14 | 79.3% | -5.375 | -1173.77 |  |
|  | Swiss Model | -3.14 | 86.70% | -1.536 | -1294.89 | Protein argonaute-1 – 4kxt.1.A (0.94) |
| BdAGO2 | CPH | -5.48 | 81.1% | -4.973 | -1678.64 |  |
|  | Swiss Model | -3.28 | 87.00% | -1.786 | -1869.75 | Protein argonaute-2 – 4f3t.1.A (0.87) |
| BdAGO3 | CPH | -5.67 | 79.5% | -5.237 | -1679.2 |  |
|  | Swiss Model | -3.32 | 86.50% | -2.586 | -1706.52 | Protein argonaute-2 – 4w5n.1.A (0.82) |
| BdAGO4 | CPH | -7.65 | 79.4% | -5.321 | -1552.69 |  |
|  | Swiss Model | -3.64 | 85.30% | -2.321 | -1847.83 | Protein argonaute-2 – 4w5n.1.A (0.91) |
| BdAGO5 | CPH | -4.74 | 81.6% | -4.936 | -1783.77 |  |
|  | Swiss Model | -2.85 | 88.40% | -1.206 | -1920.25 | Protein argonaute-2 – 4z4f.1.A (0.94) |
| BdAGO6 | CPH | -4.33 | 82.30% | -4.94 | -1769.1 |  |
|  | Swiss Model | -2.67 | 87.50% | -2.23 | -1889.91 | Protein argonaute-2 – 4w5n.1.A (0.96) |
| BdAGO7 | CPH | -4.99 | 80.8% | -4.814 | -1753.88 |  |
|  | Swiss Model | -3.35 | 86.20% | -2.068 | -1867.99 | Protein argonaute-2 – 4w5n.1.A (0.82) |
| BdAGO8 | CPH | -4.94 | 82.7% | -5.319 | -1777.54 |  |
|  | Swiss Model | -3.38 | 86.10% | -2.074 | -1930.16 | Protein argonaute-2 – 4w5n.1.A (0.79) |
| BdAGO9 | CPH | -3.61 | 84.6% | -4.933 | -1803.08 |  |
|  | Swiss Model | -2.77 | 87.00% | -1.643 | -1928.83 | Protein argonaute-2 – 4w5n.1.A (0.87) |
| BdAGO10 | CPH | -3.98 | 83.7% | -4.673 | -1766.73 |  |
|  | Swiss Model | -2.68 | 88.20% | -1.34 | -1903.52 | Protein argonaute-2 - 4z4f.1.A (0.80) |
| BdAGO11 | CPH | -3.77 | 84.1% | -4.942 | -1778.22 |  |
|  | Swiss Model | -2.46 | 88.20% | -1.749 | -1796.97 | Protein argonaute-2 – 4w5n.1.A (0.80) |
| BdAGO12 | CPH | -4.16 | 83.6% | -5.042 | -1806.27 |  |
|  | Swiss Model | -2.98 | 86.20% | -1.992 | -1944.46 | Protein argonaute-1 – 4kxt.1.A (0.76) |
| BdAGO13 | CPH | -8.48 | 76.2% | -5.955 | -1502.92 |  |
|  | Swiss Model | -3.26 | 86.60% | -2.38 | -1726.87 | Protein argonaute-2 – 4w5n.1.A (0.83) |
| BdAGO14 | CPH | -5.36 | 79.8% | -5.108 | -1567.95 |  |
|  | Swiss Model | -2.63 | 89.30% | -1.744 | -1758.43 | Protein argonaute-2- 5t7b.1.B (0.75) |
| BdAGO15 | CPH | -4.42 | 83.4% | -5.035 | -1777.14 |  |
|  | Swiss Model | -3.2 | 87.40% | -1.706 | -1946.8 | Protein argonaute-2 – 4w5n.1.A (0.78) |
| BdAGO16 | CPH | -3.58 | 83.4% | -5.044 | -1806.1 |  |
|  | Swiss Model | -2.2 | 89.00% | -1.575 | -1940.92 | Protein argonaute-2- 4z4f.1.A (0.80) |

**Table S5: Validation of predicted 3D structures of AtAGO proteins. Prediction with CPH and Swiss Model. validation by QMEAN, PROCHECK, WHATCHECK and dFIRE. Last column indicates the template used for the model and coverage.**

| **Protein** | **3D Modelling software** | **QMEAN (Z-score)** | **Residues in favored region (PROCHECK)** | **Ramachandran z-score (WHATCHECK)** | **dDFIRE total energy** | **Template used (coverage)** |
| --- | --- | --- | --- | --- | --- | --- |
| AtAGO1 | CPH | -4.21 | 83.6% | -4.76 | -1789.47 |  |
|  | Swiss Model | -2.15 | 89.2% | -1.28 | -1987.42 | Protein argonaute-1- 4kre.1.A (0.79) |
| AtAGO2 | CPH | -5.47 | 80.80% | -5.282 | -1698.99 |  |
|  | Swiss Model | -3.76 | 86.3% | -1.825 | -1851.7 | Protein argonaute-2- 4w5n.1.A (0.81) |
| AtAGO3 | CPH | -4.96 | 82.0% | -5.318 | -1714.79 |  |
|  | Swiss Model | -3.23 | 87.8% | -1.902 | -1901.54 | Protein argonaute-2 - 4f3t.1.A (0.70) |
| AtAGO4 | CPH | -4.76 | 80.7% | -4.976 | -1695.86 |  |
|  | Swiss Model | -3.08 | 86.3% | -1.997 | -1896.01 | Protein argonaute-2- 4w5n.1.A (0.87) |
| AtAGO5 | CPH | -3.78 | 83.8% | -4.96 | -1767.24 |  |
|  | Swiss Model | -2.75 | 88.5% | -1.457 | -1903.68 | Protein argonaute-2- 4w5n.1.A (0.83) |
| AtAGO6 | CPH | -4.72 | 82.4% | -4.847 | -1782.18 |  |
|  | Swiss Model | -2.81 | 87.8% | -1.613 | -1878.9 | Protein argonaute-2- 5js2.1.A (0.91) |
| AtAGO7 | CPH | -5.23 | 78.9% | -5.275 | -1690.98 |  |
|  | Swiss Model | -2.81 | 88.2% | -1.472 | -1923.34 | Protein argonaute-2-4z4h.1.A (0.82) |
| AtAGO8 | CPH | -6.4 | 77.4% | -5.105 | -1569.03 |  |
|  | Swiss Model | -3.26 | 90.0% | -1.922 | -1646.43 | Protein argonaute-1-4kre.1.A (0.86) |
| AtAGO9 | CPH | -4.28 | 80.5% | -5.024 | -1685.77 |  |
|  | Swiss Model | -3.26 | 84.6% | -2.29 | -1875.11 | Protein argonaute-2- 4w5n.1.A (0.90) |
| AtAGO10 | CPH | -5.59 | 84.0% | -5.135 | -1726.53 |  |
|  | Swiss Model | -2.56 | 91.2% | -1.397 | -1954.61 | Protein argonaute-2-5t7b.1.B (0.84) |

**Table S6: Interacting proteins of BdAGOs predicted with STRING (coexpression and experimental validation only connections shown)**

| **BdAGO Protein** | **Interacts with:** | **Interacting protein description** | **Coexpression Score** | **Experimental Validation Score** | **Combined Score** | **Reference** |
| --- | --- | --- | --- | --- | --- | --- |
| BdAGO9 | BRADI1G07247.1 | Hypothetical protein | 0 | 0.486 | 0.485 | Ding et al., 2015 |
| BdAGO9 | BRADI1G10047.1 | Hypothetical protein | 0 | 0.486 | 0.485 | Ding et al., 2015 |
| BdAGO9 | BRADI1G12677.1 | homeobox protein knotted-1-like 4 | 0 | 0.486 | 0.485 | Ding et al., 2015 |
| BdAGO9 | BRADI1G12690.1 | homeobox protein knotted-1-like 4 isoform X1 | 0 | 0.486 | 0.485 | Ding et al., 2015 |
| BdAGO1; BdAGO2; BdAGO3 | BRADI1G36340.1 | 110 kDa U5 small nuclear ribonucleoprotein component CLO | 0.043 | 0.453 | 0.454 | Kallgren et al., 2014 |
| BdAGO4; BdAGO5; BdAGO6; BdAGO7; BdAGO8; BdAGO9; BdAGO10; BdAGO11; BdAGO12; BdAGO13; BdAGO14; BdAGO15; BdAGO16 |  |  | 0 |  | 0.453 |  |
| BdAGO9 | BRADI1G57607.1 | homeobox protein knotted-1-like 12 | 0 | 0.486 | 0.485 | Ding et al., 2015 |
| BdAGO9 | BRADI2G14890.1 | GATA transcription factor 15 | 0.047 | 0.481 | 0.484 | Ding et al., 2015 |
| BdAGO1; BdAGO2; BdAGO3 | BRADI2G30160.1 | GTP binding/ Transcription factor | 0.043 | 0.453 | 0.454 | Kallgren et al., 2014 |
| BdAGO4; BdAGO5; BdAGO6; BdAGO7; BdAGO8; BdAGO9; BdAGO10; BdAGO11; BdAGO12; BdAGO13; BdAGO14; BdAGO15; BdAGO16 |  |  | 0 |  | 0.453 |  |
| BdAGO9 | BRADI2G45750.1 | GATA transcription factor 19 | 0.047 | 0.481 | 0.484 | Ding et al., 2015 |
| BdAGO1; BdAGO2; BdAGO3; BdAGO4; BdAGO5; BdAGO6; BdAGO7; BdAGO8; BdAGO9; BdAGO10; BdAGO11; BdAGO12; BdAGO13; BdAGO14; BdAGO15; BdAGO16 | BRADI4G45065.1 | DNA-directed RNA polymerase V subunit 1 | 0.045 | 0.626 | 0.627 | Zhong et al., 2015 |

**Table S7: Prediction of location of BdAGO-like proteins. as done by PSI (Plant Subcellular-localization Integrative predictor). Displayed are the scores (0-1) representing the confidence the protein is present in a cellular part. All scores have p-values < 0.01.**

| **Protein name** | **Predicted location** | **Score** |
| --- | --- | --- |
| BdAGO1 | cytosol | 0.59008 |
| BdAGO2 | cytosol | 0.58123 |
| BdAGO3 | nucleus | 0.56674 |
| BdAGO4 | cytosol | 0.53687 |
| BdAGO5 | cytosol | 0.56023 |
| BdAGO6 | cytosol | 0.56837 |
| BdAGO7 | plastid | 0.47348 |
| BdAGO8 | cytosol | 0.65765 |
| BdAGO9 | cytosol | 0.647 |
| BdAGO10 | cytosol | 0.48456 |
| BdAGO11 | cytosol | 0.60782 |
| BdAGO12 | cytosol | 0.56826 |
| BdAGO13 | cytosol | 0.6786 |
| BdAGO14 | nucleus | 0.51582 |
| BdAGO15 | cytosol | 0.6013 |
| BdAGO16 | cytosol | 0.36562 |

**Figure S1: 3D structure prediction of BdAGO8, as modeled by SWISS-MODEL. AtAGO7 3D structure prediction is displayed as the closest homolog in Arabidopsis. PAZ (yellow), Mid (red) and PIWI (blue) domains as predicted by SMART and PFAM displayed. The catalytic tetrad within the PIWI domain (DEDH) marked by magenta spheres. Visualization by PyMOL.**


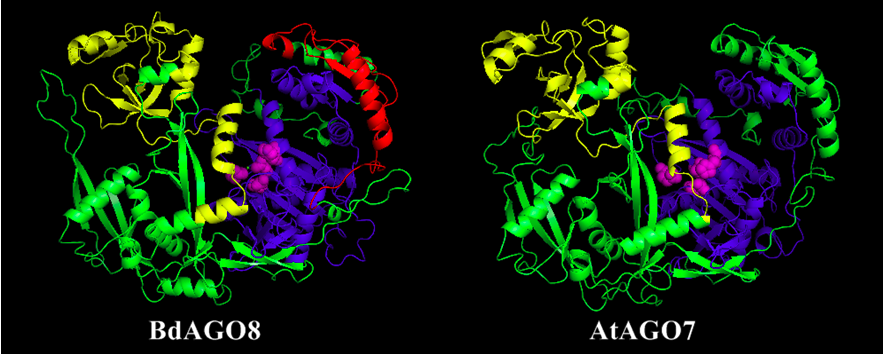


**Figure S2: Interacting proteins of BdAGO9 predicted with STRING (coexpression and experimental validation only connections valid). Pink connection: based on experimental data. Black connection: based on coexpression.**


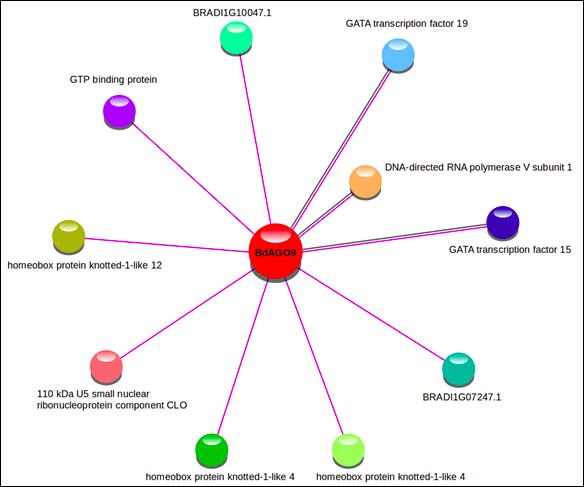

Supplement: Supplementary file 1 [file DataSheet_1.docx]
